# Estimating Short-Term and Long-Term Survival for Patients With Kidney Failure Using a Mixture Survival Model

**DOI:** 10.1016/j.xkme.2025.101232

**Published:** 2025-12-26

**Authors:** Nathan Meyer, Maxwell Donelan, Hossein Moradi Rekabdarkolaee, Brandon M. Varilek, Surachat Ngorsuraches, Patti Brooks, Jerry Schrier, Semhar Michael

**Affiliations:** 1 Department of Mathematics and Statistics, South Dakota State University, Brookings, SD; 2 Department of Business Analytics, Economics, and Information Systems, Bowling Green State University, Bowling Green, OH; 3 College of Nursing - Omaha Division, University of Nebraska Medical Center, Omaha, NE; 4 Department of Health Outcomes Research and Policy, Auburn University, Auburn, AL; 5 College of Business and Information Systems, Dakota State University, Madison, SD; 6 Avera Medical Group Nephrology, Avera McKennan Hospital & University Health Center, Sioux Falls, SD

**Keywords:** Kidney failure, time-to-event data, finite mixture modeling, survival analysis, USRDS

## Abstract

**Rationale & Objective:**

Traditional survival models assume all patients receiving kidney replacement therapy (KRT) may be grouped into one population, overlooking long-term survivors, particularly successful transplant recipients, and may fail to appreciate the disparities in minority populations. On the other hand, a mixture survival model allows for the estimation of hazard and odds ratios of all-cause mortality in patients with kidney failure undergoing either dialysis or transplantation.

**Study Design:**

This retrospective cohort study analyzed survival outcomes using a proportional hazards mixture survival model, comparing results to a traditional Cox proportional hazards model with time-varying modality of treatment.

**Setting & Participants:**

Data from the United States Renal Data System included 2,228,693 patients initiating KRT between 2000 and 2020.

**Predictors:**

Key predictors included demographics, comorbid conditions, socioeconomic status, geographic location, and rurality.

**Outcomes:**

The primary outcome was all-cause mortality. The mixture survival model distinguishes between patients’ characteristics associated with long-term survival (ie, primarily those with successful transplants) and short-term survival (ie, those at a greater risk of mortality over time, such as patients treated with dialysis).

**Analytical Approach:**

Both a Cox proportional hazards model and a proportional hazards mixture survival model were applied to all patients.

**Results:**

Findings from both models were largely consistent, but the mixture survival model revealed new insights into racial disparities. In the Cox model, American Indian individuals had an adjusted hazard ratio of 0.63 compared with White individuals (95% CI. 0.62-0.63) and 0.74 for Black individuals compared with White (95% CI, 0.74-0.74). The mixture model confirmed these trends but also showed that American Indian individuals were 1.59 times more likely to not have a long-term survival than White individuals (95% CI, 1.415-1.797) and Black individuals were 1.35 times more likely to not be in the long-term surviving group than White individuals (95% CI, 1.310-1.397). Additional disparities were observed by socioeconomic and geographic factors.

**Limitations:**

Data collected at the beginning of dialysis may not fully capture patients’ health trajectories.

**Conclusions:**

The mixture survival model provides a more comprehensive understanding of mortality disparities for patients with kidney failure receiving KRT by distinguishing between short-term and long-term survivability. The findings highlight the need for targeted interventions to improve long-term outcomes for minority patients.

## Plain-language Summary

Kidney failure affects minority groups at a high rate, but much of the current research explores a few of these groups versus a more inclusive approach. Therefore, we examined survival outcomes in over 2 million US patients with kidney failure who received dialysis or a kidney transplant. The results showed that Black and American Indian patients with kidney failure had better short-term survival but worse long-term survival odds as compared with White patients, and disparities were linked to age at first dialysis, socioeconomic and geographic factors. By using the mixture survival model, the study provided new insights into who is more likely to live longer after starting dialysis or receiving a transplant. This provides a base of evidence to develop interventions targeted at improving long-term survival and health outcomes for underserved and isolated patient populations with kidney failure.

More than 800,000 people in the United States live with kidney failure (KF).^1^ Although patients with KF undergo either hemodialysis 3 times a week for a 3-4 hours each treatment,^2^ or daily peritoneal dialysis,^3^ the preferred treatment is kidney transplantation.^4^ However, access to transplant vary among different patient populations due to age,^5^ comorbidities,^6^ and social factors.^7^ The current literature on patients with KF is heavily focused on select racial and ethnic groups (eg, non-Hispanic White and Black) due to the significant survival disparities among them.^8^, ^9^, ^10^, ^11^, ^12^, ^13^, ^14^ Literature also indicated that various social determinants of health (SDOH), such as geographical isolation would greatly impact the risk of all-cause mortality for patients receiving dialysis.^15^^,^^16^ However, these obstacles had a mixed effect on mortality.

Furthermore, patients who receive a successful kidney transplant may be considered to be within a subpopulation that is characterized as having greater survival, as they no longer require dialysis treatment. Historically, a survival analysis, eg, Cox proportional hazards model (henceforth referred to as CPH model), was primarily used to draw inferences from datasets that only involved patients on dialysis because the survival outcomes for those who did or did not receive a transplant were widely different, and it would be inappropriate to include both groups in this simple survival analysis.^17^, ^18^, ^19^ Time-varying components within a survival model have been used to account for these differences.^20^ In this paper, we consider a CPH model with a time-varying covariate for modality (including transplant status) as patients are able to switch between treatment types over time. We compare this CPH model to a mixture survival model (MSM) to account for two latent subpopulations within the chronic kidney disease patient data obtained from the United States Renal Data System (USRDS).

The 2 (unknown at the start of dialysis) subpopulations include the 2 distinct groups of patients who will or will not receive a successful kidney transplant and are able to be modeled jointly using the MSM. Further, the MSM is able to identify both the probability of long-term survival and short-term hazard for patients with KF. Specifically, a MSM is commonly used in oncology research due to the advances in treatments in which some patients are labeled as cured and will have different survival outcomes than those who are not.^21^^,^^22^ The movement between the 2 subpopulations is not predetermined because those with unsuccessful transplants need dialysis, and those on dialysis might get a transplant throughout the observation period. To our knowledge, only 2 studies used MSM with dialysis patients. However, one study focused on patients undergoing hemodialysis, while another one analyzed dialysis patients with a specific complication.^23^^,^^24^

Hence, the objective of this study was to estimate the survival probabilities for individuals receiving KRT, including patients on dialysis and patients with a kidney transplant, in the United States, using a proportional hazards MSM and accounting for various covariates, eg, demographics and SDOH.

## Methods

### Dataset

**Figure 1:**
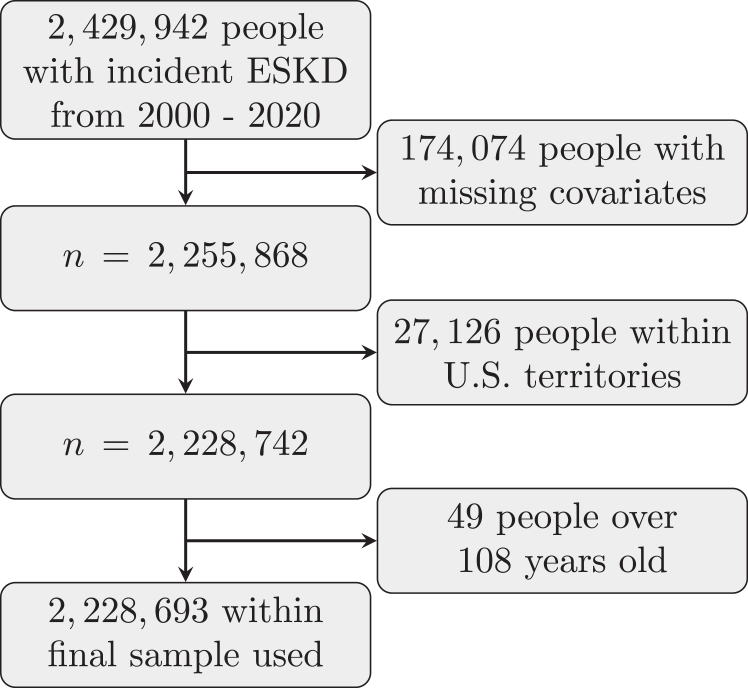
Flow chart of the sample selection criterion.

Data provided by the USRDS consists of 2,429,942 unique individuals who initiated KRT (dialysis/transplantation) from 2000 to 2020.^25^^,^^26^ Our analysis included follow-up data through 2021 of these patients. The date of the last recorded death was used as the cut-off to perform right-censoring in the survival analysis. The study was reviewed by the institutional board and was found exempt. The need for informed consent was waived due to deidentified information. After investigating each covariate, the observations with missing values for any covariates were removed from the final sample dataset. Fig 1 shows this process, resulting in a final dataset with 2,228,693 patients. This final dataset focuses on those without preemptive transplants. All-cause mortality was unobserved for 658,655 patients (considered to have a censored outcome); hence, the event of interest (all-cause mortality) was observed for the other 1,570,038 patients. This is about 70% mortality within the study period and deaths being reported for anyone with transplants and dialysis. We observed that the median survival time was about 3 years from the start of dialysis for the overall data and around 2.5 and 10 years for dialysis patients versus those with at least one transplantation, respectively. The time variable was determined by subtracting either the day when a patient’s all-cause mortality was declared or when a patient was right censored from the day when the same individual first enrolled in KRT. The USRDS data included a list of 22 comorbid conditions. Most of these conditions were summarized using the Liu comorbidity index,^27^ a modification of the Charlson comorbidity index.^28^ The remaining covariates included age, sex, race, ethnicity, insurance status, rurality, employment status, region, dialysis treatment type, alcohol dependence, drug dependence, and tobacco use. The 34 insurance options found within the USRDS data were categorized into 9 groups. Some groups might not be immediately intuitive. For instance, the Department of Veteran Affairs Plus (DVA+) indicated all individuals with either DVA insurance alone or DVA insurance along with any other insurance coverage. Furthermore, the Medicare and Employer category specifies those with only both Medicare and employer insurance. Similarly, for the Medicare and Medicaid category. Finally, Multiple otherwise would designate a person with more than one insurance coverage, but that did not fall into any previously discussed category. Further discussion of these insurance groups can be found in the supplementary material.

### Statistical Analyses

We conducted descriptive analyses of all variables for overall patients and patients with and without transplants. The statistical model employed in this paper is the mixture survival model, specifically a mixture with 2 components with one restricted to having a uniform density. This model is commonly known as a mixture cure model and appears mostly in oncology literature. Specifically, MSM has the following form: S(t;x,z)=π(z)S(t;x)+1−π(z), where the observed data includes $d_{j}=\left(t_{j},x_{j}^{\prime },z_{j}^{\prime },\delta_{j}\right)$ for j=1,…,N, with N representing the total number of individuals and $\delta_{j}$ representing whether the event of interest is observed or censoring has occurred. In this work we considered the mixing proportions to be logistic function given by $\pi \left(z\right)=\frac{\exp\left\{b^{\prime }z\right\}}{1+\exp\left\{b^{\prime }z\right\}}$, where 1-π(z) represents the odds of long-term survival. For the survival function we apply a proportional hazard model given by $s\left(t;x\right)=s_{0}{\left(t\right)}^{\left\{\exp\left(\beta x\right)\right\}}$, where $s_{0}\left(t\right)$ is the baseline survival function for the first subpopulation.

For comparison, a CPH model was fitted to the full set of data. However, since individuals may change modality (including transplant status) during the study period, the modality variable was considered time-dependent within the CPH model. Furthermore, an interaction between modality and time was also included within the CPH model to allow for a time-dependent hazard ratio. We also developed an MSM using only baseline information for dialysis type, as the MSM already captures time-dependent effects for all variables in terms of short-term and long-term survivability (transplant status was included separately from the modality variable within the MSM).

Both the CPH model and MSM were multivariable-adjusted. Furthermore, both models used all patients, including those who were dialysis patients or had received a kidney transplant. The MSM provided 2 inferences, including the short-term and long-term effects of all explanatory variables. The short-term effect represents the hazard of all-cause mortality for those within the group of individuals that may be characterized as having a lower survivability (eg, those still receiving dialysis treatment) as opposed to the group characterized as having a higher survivability (eg, those that will receive a successful kidney transplant). On the contrary, the long-term effect refers to the odds of an individual being within the lower survivability group.^29^ In other words, higher hazard indicates lower short-term survivability, and higher odds represent lower long-term survivability. All covariates, except Liu’s comorbidity index, were treated as categorical variables. R Statistical Software version 4.3.2. was used for all statistical analyses.^30^

**Figure 2:**
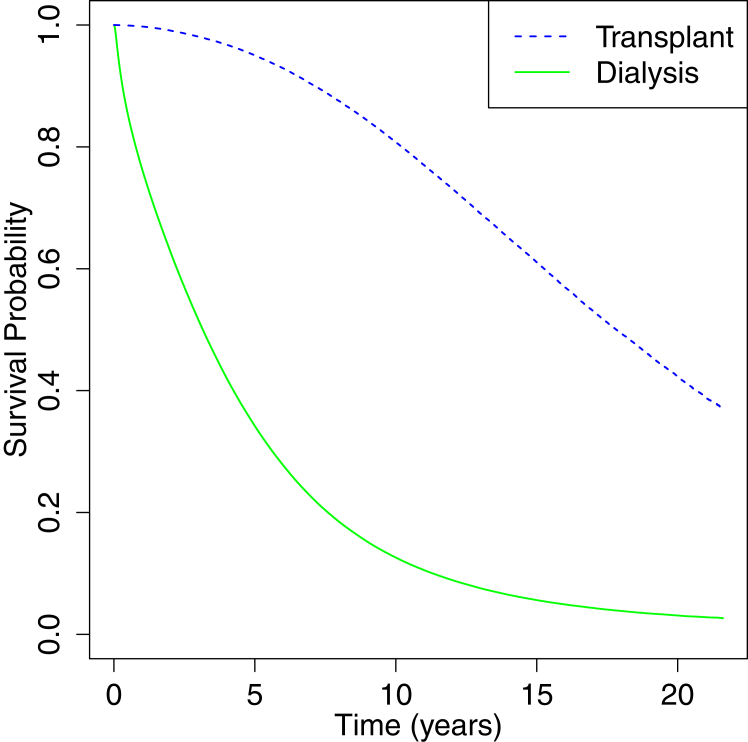
Kaplan-Meier survival plot of the USRDS dataset partitioned by transplant status.

The appropriateness of using the MSM may be addressed using both biological and empirical evidence. Specifically, the MSM should be used only when strong evidence to do so arises.^31^ There are five main concepts to consider for when a dataset would benefit from the application of an MSM: biological evidence, empirical evidence, large sample, long-term follow-up, and non-excessive censoring.^32^ As mentioned previously, individuals who have received a successful transplant would no longer be on dialysis and have greater survival indicating biological evidence for using an MSM. Empirical evidence also supports the use of such a model as about 11.0% of the ∼2.2 million people within the dataset have had at least one transplant and this would contain successful transplant recipients. Fig 2 displays a Kaplan-Meier survival plot of the USRDS dataset partitioned by transplant status. This plot indicates a vast difference between survival outcomes among transplant recipients and individuals who only have received dialysis giving additional empirical evidence for the use of an MSM. Further discussion on the appropriateness of MSM of this study may be found within the supplementary material.

## Results

**Figure 3:**
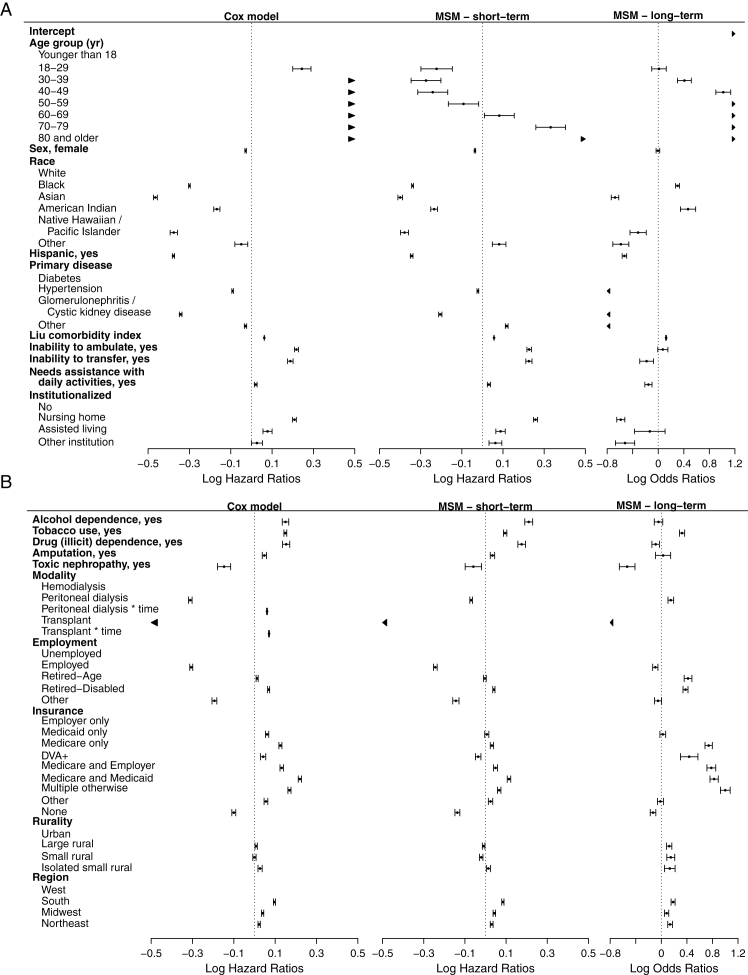
Forest plot of the multivariable-adjusted parameter estimates and corresponding confidence intervals for the two settings considered. An arrow indicates that the estimate is outside the limits of the x-axis used within the display. Unlike the Cox model, transplant status is separate from modality within the MSM and has a reference group of no transplant.

Table 1 shows the characteristics of the patients analyzed along with details partitioned by transplant status. Table 2 shows the parameter estimates from the CPH model, while Table 3 presents the parameter estimates from the MSM. All parameter estimates shown in both tables are fully adjusted for all variables included (a multivariate analysis). The adjusted hazard ratios (henceforth indicated by HR) were estimated in the CPH model. On the other hand, the MSM models’ both HRs and adjusted odds ratios (henceforth indicated by OR) were estimated. Specifically, HRs were associated with the short-term portion of MSM, while the ORs were associated with the long-term portion of the MSM. Furthermore, a forest plot representing the results from CPH model and MSM is provided in Fig 3 for ease of comparison and interpretation. For example, the MSM showed an overall trend of an increasing OR as age increased. A similar trend may be found for the HRs within the CPH model. On the other hand, the MSM showed that the HR first decreased with age, followed by an increasing trend.

**Table 1:** Baseline Characteristics of the Patients With Kidney Failure From USRDS Dataset

Characteristic	Overall^a^	No Transplant^a^	At Least 1 Transplant^a^
	N = 2,228,693	n = 1,982,698	n = 245,995
**Age (y)**	62.8 ± 15.5	64.8 ± 14.3	46.9 ± 15.2
**Age group (y)**			
Younger than 18	13,120 (0.6%)	3,219 (0.2%)	9,901 (4.0%)
18-29	47,343 (2.1%)	24,905 (1.3%)	22,438 (9.1%)
30-39	106,781 (4.8%)	70,187 (3.5%)	36,594 (14.9%)
40-49	217,583 (9.8%)	165,348 (8.3%)	52,235 (21.2%)
50-59	405,556 (18.2%)	341,539 (17.2%)	64,017 (26.0%)
60-69	551,288 (24.7%)	502,470 (25.3%)	48,818 (19.8%)
70-79	536,313 (24.1%)	524,727 (26.5%)	11,586 (4.7%)
80 and older	350,709 (15.7%)	350,303 (17.7%)	406 (0.2%)
**Sex**			
Male	1,262,637 (56.7%)	1,110,227 (56.0%)	152,410 (62.0%)
Female	966,056 (43.3%)	872,471 (44.0%)	93,585 (38.0%)
**Race**			
White	1,469,364 (65.9%)	1,312,148 (66.2%)	157,216 (63.9%)
Black	620,871 (27.9%)	551,767 (27.8%)	69,104 (28.1%)
Asian	83,555 (3.7%)	69,988 (3.5%)	13,567 (5.5%)
American Indian	24,315 (1.1%)	21,974 (1.1%)	2,341 (1.0%)
Native Hawaiian /Pacific Islander	22,133 (1.0%)	19,522 (1.0%)	2,611 (1.1%)
Other	8,455 (0.4%)	7,299 (0.4%)	1,156 (0.5%)
**Hispanic**			
No	1,922,932 (86.3%)	1,718,212 (86.7%)	204,720 (83.2%)
Yes	305,761 (13.7%)	264,486 (13.3%)	41,275 (16.8%)
**Primary disease**			
Diabetes	1,037,380 (46.5%)	957,355 (48.3%)	80,025 (32.5%)
Hypertension	644,080 (28.9%)	586,160 (29.6%)	57,920 (23.5%)
Glomerulonephritis or Cystic kidney disease	226,088 (10.1%)	150,761 (7.6%)	75,327 (30.6%)
Other	321,145 (14.4%)	288,422 (14.5%)	32,723 (13.3%)
**Liu comorbidity index**	2.7 ± 2.5	2.8 ± 2.6	1.1 ± 1.7
**Inability to ambulate**			
No	2,082,718 (93.5%)	1,839,109 (92.8%)	243,609 (99.0%)
Yes	145,975 (6.5%)	143,589 (7.2%)	2,386 (1.0%)
**Inability to transfer**			
No	2,155,052 (96.7%)	1,910,084 (96.3%)	244,968 (99.6%)
Yes	73,641 (3.3%)	72,614 (3.7%)	1,027 (0.4%)
**Needs assistance with daily activities**			
No	1,997,597 (89.6%)	1,757,990 (88.7%)	239,607 (97.4%)
Yes	231,096 (10.4%)	224,708 (11.3%)	6,388 (2.6%)
**Institutionalized**			
No	2,083,006 (93.5%)	1,838,910 (92.7%)	244,096 (99.2%)
Nursing home	123,391 (5.5%)	122,020 (6.2%)	1,371 (0.6%)
Assisted living	11,890 (0.5%)	11,705 (0.6%)	185 (0.1%)
Other institution	10,406 (0.5%)	10,063 (0.5%)	343 (0.1%)
**Alcohol dependence**			
No	2,192,881 (98.4%)	1,948,987 (98.3%)	243,894 (99.1%)
Yes	35,812 (1.6%)	33,711 (1.7%)	2,101 (0.9%)
**Tobacco use**			
No	2,088,788 (93.7%)	1,852,054 (93.4%)	236,734 (96.2%)
Yes	139,905 (6.3%)	130,644 (6.6%)	9,261 (3.8%)
**Drug (illicit) dependence**			
No	2,199,631 (98.7%)	1,955,044 (98.6%)	244,587 (99.4%)
Yes	29,062 (1.3%)	27,654 (1.4%)	1,408 (0.6%)
**Amputation**			
No	2,169,109 (97.3%)	1,925,927 (97.1%)	243,182 (98.9%)
Yes	59,584 (2.7%)	56,771 (2.9%)	2,813 (1.1%)
**Toxic nephropathy**			
No	2,221,206 (99.7%)	1,976,043 (99.7%)	245,163 (99.7%)
Yes	7,487 (0.3%)	6,655 (0.3%)	832 (0.3%)
**Modality**			
Hemodialysis	2,035,286 (91.3%)	1,839,725 (92.8%)	195,561 (79.5%)
Peritoneal dialysis	193,407 (8.7%)	142,973 (7.2%)	50,434 (20.5%)
**Employment**			
Unemployed	564,112 (25.3%)	496,559 (25.0%)	67,553 (27.5%)
Employed	235,437 (10.6%)	155,418 (7.8%)	80,019 (32.5%)
Retired-Age	891,167 (40.0%)	860,052 (43.4%)	31,115 (12.6%)
Retired-Disabled	462,421 (20.7%)	420,255 (21.2%)	42,166 (17.1%)
Other	75,556 (3.4%)	50,414 (2.5%)	25,142 (10.2%)
**Insurance**			
Employer only	288,736 (13.0%)	200,863 (10.1%)	87,873 (35.7%)
Medicaid only	266,451 (12.0%)	235,632 (11.9%)	30,819 (12.5%)
Medicare only	558,546 (25.1%)	530,039 (26.7%)	28,507 (11.6%)
DVA+	43,197 (1.9%)	40,008 (2.0%)	3,189 (1.3%)
Medicare and Employer	149,707 (6.7%)	134,167 (6.8%)	15,540 (6.3%)
Medicare and Medicaid	291,050 (13.1%)	274,824 (13.9%)	16,226 (6.6%)
Multiple otherwise	353,353 (15.9%)	333,344 (16.8%)	20,009 (8.1%)
Other	135,965 (6.1%)	113,248 (5.7%)	22,717 (9.2%)
None	141,688 (6.4%)	120,573 (6.1%)	21,115 (8.6%)
**Rurality**			
Urban	1,870,585 (83.9%)	1,658,041 (83.6%)	212,544 (86.4%)
Large rural	199,543 (9.0%)	180,960 (9.1%)	18,583 (7.6%)
Small rural	100,806 (4.5%)	91,721 (4.6%)	9,085 (3.7%)
Isolated small rural	57,759 (2.6%)	51,976 (2.6%)	5,783 (2.4%)
**Region**			
West	462,484 (20.8%)	409,051 (20.6%)	53,433 (21.7%)
South	928,422 (41.7%)	832,984 (42.0%)	95,438 (38.8%)
Midwest	463,525 (20.8%)	410,366 (20.7%)	53,159 (21.6%)
Northeast	374,262 (16.8%)	330,297 (16.7%)	43,965 (17.9%)

a *Note:* Mean ± SD; n (%).

**Table 2:** Multivariable-Adjusted Parameter Estimates for the Cox Regression Model for All-Cause Mortality for Patients With Kidney Failure

Characteristic	Coefficient estimates	s.e.	Hazard ratio (95% CI)	*P*
**Age group (y)**				
Younger than 18	(ref)			
18-29	0.2441	0.0222	1.28 (1.22-1.33)	< 0.001
30-39	0.5811	0.0210	1.79 (1.72-1.86)	< 0.001
40-49	0.8579	0.0207	2.36 (2.26-2.46)	< 0.001
50-59	1.1250	0.0206	3.08 (2.96-3.21)	< 0.001
60-69	1.3628	0.0206	3.91 (3.75-4.07)	< 0.001
70-79	1.6626	0.0207	5.27 (5.06-5.50)	< 0.001
80 and older	2.0382	0.0207	7.68 (7.37-8.00)	< 0.001
**Sex,** female	−0.0280	0.0017	0.97 (0.97-0.98)	< 0.001
**Race**				
White	(ref)			
Black	−0.3000	0.0020	0.74 (0.74-0.74)	< 0.001
Asian	−0.4649	0.0049	0.63 (0.62-0.63)	< 0.001
American Indian	−0.1673	0.0078	0.85 (0.83-0.86)	< 0.001
Native Hawaiian/Pacific Islander	−0.3758	0.0091	0.69 (0.68-0.70)	< 0.001
Other	−0.0490	0.0135	0.95 (0.92-0.98)	0.002
**Hispanic,** yes	−0.3773	0.0028	0.69 (0.68-0.69)	< 0.001
**Primary disease**				
Diabetes	(ref)			
Hypertension	−0.0911	0.0020	0.91 (0.91-0.92)	< 0.001
Glomerulonephritis/Cystic kidney disease	−0.3425	0.0033	0.71 (0.71-0.71)	< 0.001
Other	−0.0287	0.0025	0.97 (0.97-0.98)	< 0.001
**Liu comorbidity index**	0.0623	0.0003	1.06 (1.06-1.06)	< 0.001
**Inability to ambulate,** yes	0.2178	0.0041	1.24 (1.23-1.25)	< 0.001
**Inability to transfer,** yes	0.1884	0.0053	1.21 (1.19-1.22)	< 0.001
**Needs assistance with daily activities,** yes	0.0207	0.0031	1.02 (1.01-1.03)	< 0.001
**Institutionalized**				
No	(ref)			
Nursing home	0.2082	0.0038	1.23 (1.22-1.24)	< 0.001
Assisted living	0.0779	0.0102	1.08 (1.06-1.10)	< 0.001
Other institution	0.0268	0.0115	1.03 (1.00-1.05)	0.05
**Alcohol dependence,** yes	0.1498	0.0066	1.16 (1.14-1.18)	< 0.001
**Tobacco use,** yes	0.1491	0.0034	1.16 (1.15-1.17)	< 0.001
**Drug (illicit) dependence,** yes	0.1533	0.0077	1.17 (1.15-1.19)	< 0.001
**Amputation,** yes	0.0478	0.0049	1.05 (1.04-1.06)	< 0.001
**Toxic nephropathy,** yes	−0.1473	0.0145	0.86 (0.84-0.89)	< 0.001
**Modality**				
Hemodialysis	(ref)			
Peritoneal dialysis	−0.3107	0.0046	0.73 (0.73-0.74)	< 0.001
Peritoneal dialysis time	0.0607	0.0011	1.06 (1.06-1.06)	< 0.001
Transplant	−1.8829	0.0113	0.15 (0.15-0.16)	< 0.001
Transplant time	0.0704	0.0012	1.07 (1.07-1.08)	< 0.001
**Employment**				
Unemployed	(ref)			
Employed	−0.3061	0.0039	0.74 (0.73-0.74)	< 0.001
Retired-age	0.0126	0.0025	1.01 (1.01-1.02)	< 0.001
Retired-disabled	0.0684	0.0025	1.07 (1.06-1.08)	< 0.001
Other	−0.1940	0.0059	0.82 (0.81-0.83)	< 0.001
**Insurance**				
Employer only	(ref)			
Medicaid only	0.0609	0.0040	1.06 (1.05-1.07)	< 0.001
Medicare only	0.1254	0.0036	1.13 (1.13-1.14)	< 0.001
DVA+	0.0418	0.0065	1.04 (1.03-1.06)	< 0.001
Medicare and Employer	0.1316	0.0042	1.14 (1.13-1.15)	< 0.001
Medicare and Medicaid	0.2195	0.0038	1.25 (1.24-1.25)	< 0.001
Multiple otherwise	0.1693	0.0038	1.18 (1.18-1.19)	< 0.001
Other	0.0553	0.0045	1.06 (1.05-1.07)	< 0.001
None	−0.1003	0.0047	0.91 (0.90-0.91)	< 0.001
**Rurality**				
Urban	(ref)			
Large rural	0.0081	0.0028	1.01 (1.00-1.01)	0.01
Small rural	−0.0003	0.0038	1.00 (0.99-1.01)	0.95
Isolated small rural	0.0269	0.0049	1.03 (1.02-1.04)	< 0.001
**Region**				
West	(ref)			
South	0.0962	0.0024	1.10 (1.10-1.11)	< 0.001
Midwest	0.0392	0.0026	1.04 (1.03-1.05)	< 0.001
Northeast	0.0226	0.0027	1.02 (1.02-1.03)	< 0.001

*Note:* Modality is a time-dependent variable and includes time interactions denoted by time.

**Table 3:** Multivariable-Adjusted Parameter Estimates for the Mixture Survival Model for Patients With Kidney Failure

Characteristic	Coefficient Estimates	s.e.	Odds Ratio (95% CI)	*P*
**Long-term effects**				
**Intercept**	1.5854	0.0681	4.88 (4.27-5.58)	< 0.001
**Age group (y)**				
Younger than 18	(ref)			
18-29	0.0117	0.0580	1.01 (0.90-1.13)	0.84
30-39	0.4088	0.0553	1.50 (1.35-1.68)	< 0.001
40-49	1.0181	0.0594	2.77 (2.46-3.11)	< 0.001
50-59	1.5998	0.0576	4.95 (4.42-5.54)	< 0.001
60-69	2.0480	0.0571	7.75 (6.93-8.67)	< 0.001
70-79	2.5912	0.0603	13.35 (11.86-15.02)	< 0.001
80 and older	3.4683	0.0721	32.08 (27.86-36.95)	< 0.001
**Sex, female**	−0.0053	0.0143	0.99 (0.97-1.02)	0.71
**Race**				
White	(ref)			
Black	0.3023	0.0164	1.35 (1.31-1.40)	< 0.001
Asian	−0.6768	0.0301	0.51 (0.48-0.54)	< 0.001
American Indian	0.4665	0.0611	1.59 (1.42-1.80)	< 0.001
Native Hawaiian/Pacific Islander	−0.3154	0.0648	0.73 (0.64-0.83)	< 0.001
Other	−0.5854	0.0640	0.56 (0.49-0.63)	< 0.001
**Hispanic, yes**	−0.5295	0.0179	0.59 (0.57-0.61)	< 0.001
**Primary disease**				
Diabetes	(ref)			
Hypertension	−1.2989	0.0263	0.27 (0.26-0.29)	< 0.001
Glomerulonephritis/Cystic kidney disease	−1.3087	0.0231	0.27 (0.26-0.28)	< 0.001
Other	−1.7385	0.0267	0.18 (0.17-0.18)	< 0.001
**Liu comorbidity index**	0.1231	0.0039	1.13 (1.12-1.14)	< 0.001
**Inability to ambulate, yes**	0.0712	0.0405	1.07 (0.99-1.16)	0.08
**Inability to transfer, yes**	−0.1823	0.0548	0.83 (0.75-0.93)	0.001
**Needs assistance with daily activities, yes**	−0.1539	0.0287	0.86 (0.81-0.91)	< 0.001
**Institutionalized**				
No	(ref)			
Nursing home	−0.5866	0.0323	0.56 (0.52-0.59)	< 0.001
Assisted living	−0.1325	0.1220	0.88 (0.69-1.11)	0.28
Other institution	−0.5200	0.0770	0.59 (0.51-0.69)	< 0.001
**Alcohol dependence, yes**	−0.0442	0.0337	0.96 (0.90-1.02)	0.19
**Tobacco use, yes**	0.3240	0.0209	1.38 (1.33-1.44)	< 0.001
**Drug (illicit) dependence, yes**	−0.0884	0.0300	0.92 (0.86-0.97)	0.003
**Amputation, yes**	0.0273	0.0604	1.03 (0.91-1.16)	0.65
**Toxic nephropathy, yes**	−0.5349	0.0625	0.59 (0.52-0.66)	< 0.001
**Modality, peritoneal dialysis**	0.1485	0.0228	1.16 (1.11-1.21)	< 0.001
**Transplant, at least 1**	−1.2711	0.0215	0.28 (0.27-0.29)	< 0.001
**Employment**				
Unemployed	(ref)			
Employed	−0.0940	0.0211	0.91 (0.87-0.95)	< 0.001
Retired-age	0.4181	0.0312	1.52 (1.43-1.61)	< 0.001
Retired-disabled	0.3781	0.0197	1.46 (1.40-1.52)	< 0.001
Other	−0.0509	0.0277	0.95 (0.90-1.00)	0.07
**Insurance**				
Employer only	(ref)			
Medicaid only	0.0227	0.0230	1.02 (0.98-1.07)	0.32
Medicare only	0.7411	0.0291	2.10 (1.98-2.22)	< 0.001
DVA+	0.4361	0.0696	1.55 (1.35-1.77)	< 0.001
Medicare and Employer	0.7825	0.0351	2.19 (2.04-2.34)	< 0.001
Medicare and Medicaid	0.8263	0.0329	2.29 (2.14-2.44)	< 0.001
Multiple otherwise	1.0009	0.0388	2.72 (2.52-2.94)	< 0.001
Other	−0.0147	0.0239	0.98 (0.94-1.03)	0.54
None	−0.1300	0.0221	0.88 (0.84-0.92)	< 0.001
**Rurality**				
Urban	(ref)			
Large rural	0.1236	0.0211	1.13 (1.09-1.18)	< 0.001
Small rural	0.1479	0.0316	1.16 (1.09-1.23)	< 0.001
Isolated small rural	0.1316	0.0424	1.14 (1.05-1.24)	0.002
**Region**				
West	(ref)			
South	0.1842	0.0160	1.20 (1.17-1.24)	< 0.001
Midwest	0.0829	0.0168	1.09 (1.05-1.12)	< 0.001
Northeast	0.1376	0.0190	1.15 (1.10-1.19)	< 0.001

**Characteristic**	**Coefficient estimates**	**s.e.**	**Hazard ratio (95% CI)**	***P***
**Short-term effects**				
**Age group (y)**				
Younger than 18	(ref)			
18-29	−0.2226	0.0390	0.80 (0.74-0.86)	< 0.001
30-39	−0.2741	0.0370	0.76 (0.71-0.82)	< 0.001
40-49	−0.2415	0.0372	0.79 (0.73-0.84)	< 0.001
50-59	−0.0919	0.0376	0.91 (0.85-0.98)	0.02
60-69	0.0821	0.0370	1.09 (1.01-1.17)	0.03
70-79	0.3315	0.0368	1.39 (1.30-1.50)	< 0.001
80 and older	0.6837	0.0370	1.98 (1.84-2.13)	< 0.001
**Sex,** female	−0.0364	0.0018	0.96 (0.96-0.97)	< 0.001
**Race**				
White	(ref)			
Black	−0.3395	0.0023	0.71 (0.71-0.71)	< 0.001
Asian	−0.3992	0.0058	0.67 (0.66-0.68)	< 0.001
American Indian	−0.2344	0.0084	0.79 (0.78-0.80)	< 0.001
Native Hawaiian/Pacific Islander	−0.3785	0.0099	0.69 (0.67-0.70)	< 0.001
Other	0.0812	0.0169	1.08 (1.05-1.12)	< 0.001
**Hispanic, yes**	−0.3438	0.0033	0.71 (0.70-0.71)	< 0.001
**Primary disease**				
Diabetes	(ref)			
Hypertension	−0.0221	0.0021	0.98 (0.97-0.98)	< 0.001
Glomerulonephritis/Cystic kidney disease	−0.2047	0.0040	0.81 (0.81-0.82)	< 0.001
Other	0.1179	0.0034	1.12 (1.12-1.13)	< 0.001
**Liu comorbidity index**	0.0565	0.0014	1.06 (1.06-1.06)	< 0.001
**Inability to ambulate, yes**	0.2271	0.0056	1.25 (1.24-1.27)	< 0.001
**Inability to transfer, yes**	0.2258	0.0077	1.25 (1.24-1.27)	< 0.001
**Needs assistance with daily activities, yes**	0.0314	0.0038	1.03 (1.02-1.04)	< 0.001
**Institutionalized**				
No	(ref)			
Nursing home	0.2576	0.0050	1.29 (1.28-1.31)	< 0.001
Assisted living	0.0882	0.0117	1.09 (1.07-1.12)	< 0.001
Other institution	0.0631	0.0159	1.06 (1.03-1.10)	< 0.001
**Alcohol dependence, yes**	0.2101	0.0098	1.23 (1.21-1.26)	< 0.001
**Tobacco use, yes**	0.0954	0.0042	1.10 (1.09-1.11)	< 0.001
**Drug (illicit) dependence, yes**	0.1756	0.0094	1.19 (1.17-1.21)	< 0.001
**Amputation, yes**	0.0337	0.0055	1.03 (1.02-1.04)	< 0.001
**Toxic nephropathy, yes**	−0.0589	0.0205	0.94 (0.91-0.98)	0.004
**Modality, peritoneal dialysis**	−0.0698	0.0031	0.93 (0.93-0.94)	< 0.001
**Transplant, at least 1**	−1.5837	0.0060	0.20 (0.20-0.21)	< 0.001
**Employment**				
Unemployed	(ref)			
Employed	−0.2440	0.0046	0.78 (0.78-0.79)	< 0.001
Retired-Age	−0.0028	0.0033	1.00 (0.99-1.00)	0.39
Retired-Disabled	0.0407	0.0028	1.04 (1.04-1.05)	< 0.001
Other	−0.1441	0.0077	0.87 (0.85-0.88)	< 0.001
**Insurance**				
Employer only	(ref)			
Medicaid only	0.0063	0.0051	1.01 (1.00-1.02)	0.22
Medicare only	0.0310	0.0041	1.03 (1.02-1.04)	< 0.001
DVA+	−0.0355	0.0068	0.96 (0.95-0.98)	< 0.001
Medicare and Employer	0.0485	0.0047	1.05 (1.04-1.06)	< 0.001
Medicare and Medicaid	0.1143	0.0044	1.12 (1.11-1.13)	< 0.001
Multiple otherwise	0.0665	0.0043	1.07 (1.06-1.08)	< 0.001
Other	0.0245	0.0055	1.02 (1.01-1.04)	< 0.001
None	−0.1368	0.0061	0.87 (0.86-0.88)	< 0.001
**Rurality**				
Urban	(ref)			
Large rural	−0.0092	0.0032	0.99 (0.98-1.00)	0.004
Small rural	−0.0208	0.0043	0.98 (0.97-0.99)	< 0.001
Isolated small rural	0.0138	0.0050	1.01 (1.00-1.02)	0.006
**Region**				
West	(ref)			
South	0.0843	0.0028	1.09 (1.08-1.09)	< 0.001
Midwest	0.0429	0.0030	1.04 (1.04-1.05)	< 0.001
Northeast	0.0301	0.0035	1.03 (1.02-1.04)	< 0.001

Within the CPH model, White patients had the highest HR, followed by the categories of other, American Indians, Black individuals, Native Hawaiians/Pacific Islanders (NHs/PIs), and then Asians. For instance, compared with White individuals, American Indian individuals had an HR of 0.85 (95% CI, 0.83-0.86) and Black individuals had HR of 0.74 (95% CI, 0.74-0.74). Similarly, the MSM showed that the category of other has the highest HR followed by White individuals, American Indians, Black individuals, NHs/PIs, and Asians. Conversely, the OR from the MSM showed that American Indians had the highest odds ratio followed by Black individuals, White individuals, Asians, the category of other, and then NHs/PIs. Specifically, American Indians had an OR of 1.59 compared with White individuals (95% CI, 1.415-1.797) and Black individuals had an OR of 1.35 compared with White individuals (95% CI, 1.310-1.397). This indicates lower likelihood of being in the long-term survival group. Additionally, Hispanic patients had a lower hazard compared to non-Hispanic patients in both models and a lower OR in the MSM.

Both the CPH model and MSM showed that patients with higher Liu comorbidity index had higher HRs whereas the MSM also showed higher ORs. Again, based on both the CPH model and MSM, patients with the inability to ambulate, inability to transfer, or needed assistance with daily activities, each individually had higher HRs than their counterparts. Similarly, institutionalized patients had higher HRs in both models. Furthermore, both the CPH model and MSM showed that patients who had an alcohol dependence, used tobacco, had an illicit drug dependence, or had amputation, each individually had higher HRs than their counterparts. Conversely, the MSM showed that patients with an inability to transfer, needed assistance with daily activities, or had an illicit drug dependence, each individually had lower ORs than their counterparts. Similarly, institutionalized patients had lower ORs. Finally, according to both models, patients with toxic nephropathy had lower HRs compared with their counterparts.

**Figure 4:**
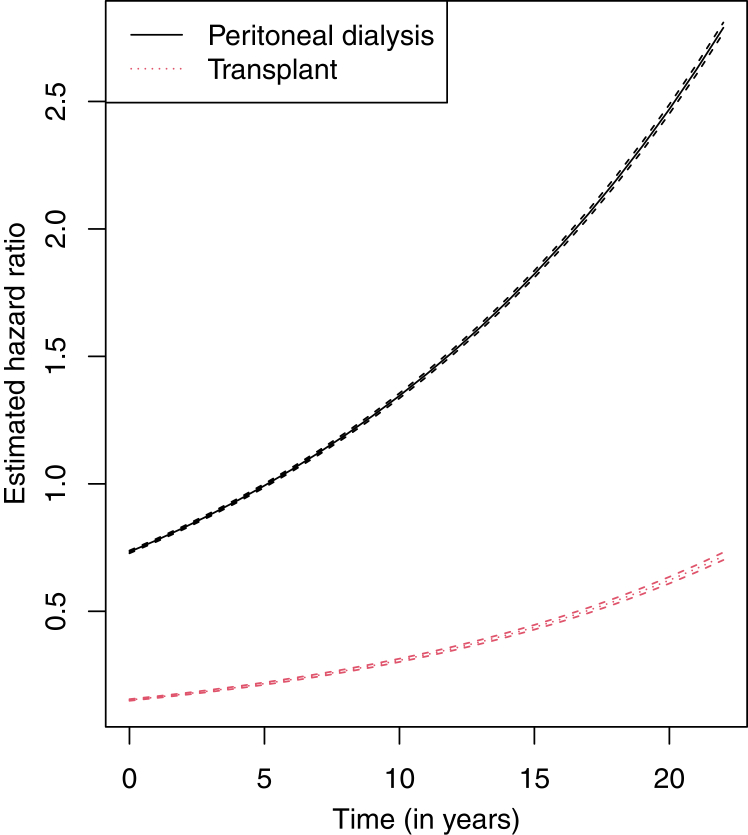
Estimated, adjusted, time-dependent hazard ratios for the modality variable (baseline is hemodialysis) and pointwise, robust 95% confidence intervals for each.

Fig 4 shows the time-dependent HRs for the modality variable along with pointwise, robust 95% CIs while keeping all other covariates constant. Within the CPH model, patients on peritoneal dialysis have a lower HR than those on hemodialysis; however, this relationship inverts around 5 years as the time-dependent HR increases past an HR of one over time. For instance, those on hemodialysis have an HR of 1.28 compared with those on peritoneal dialysis at the end of year one (95% CI, 1.275-1.293), whereas those on peritoneal dialysis have an HR of 1.27 compared with those on hemodialysis at the end of year nine (95% CI, 1.257-.276). Furthermore, patients with a transplant have a lower HR compared with those on hemodialysis. This time-dependent HR also increases over time but always remains the lowest among the modality types. For example, those with a transplant have an HR of 4.62 compared with those on hemodialysis at the end of year 5 (95% CI, 4.526-4.720). The MSM showed that patients with peritoneal dialysis had a lower HR than those with hemodialysis but a higher OR. Additionally, the MSM revealed that patients with at least one transplant had both a lower HR and OR than those with no transplant.

Compared with the unemployed group, both the CPH model and MSM showed lower HRs for the employed patients. Based on the MSM, the results of the HRs for the retired were mixed or depended upon their retirement reasons (ie, age and disability), whereas the CPH model indicated that the retired due to any reasons had higher HRs than the unemployed. The MSM also showed that the employed had lower OR compared with the unemployed. Conversely, the retired, due to either age or disability had higher ORs. Compared with employer-sponsored insurance, the MSM showed that patients with any type of insurance, except those with Medicaid only and those with no insurance had significantly higher HRs. Similarly, the CPH model showed patients with any type of insurance, except the no insurance, had a higher HR compared with the employer-sponsored insurance. The MSM revealed that patients with any type of insurance, except those with Medicaid only, those with no insurance, and those with other types of insurance had significantly higher ORs when compared with the patients with the employer-sponsored group.

Compared with patients from urban areas, the MSM showed that patients from large rural and small rural areas had lower HRs, while patients from isolated small rural areas had a higher HR. The CPH model showed that patients from large rural areas and isolated small rural areas, each had a higher HR compared with those in an urban area. The MSM showed that patients from large rural, small rural, and isolated small rural areas had higher ORs, compared with patients from urban areas. Both CPH model and MSM showed that patients from Southern, Midwestern, and Northeastern regions had higher HRs compared with patients from the Western region. Additionally, the MSM showed that patients from Southern, Midwestern, and Northeastern regions had higher ORs compared with patients from the Western region.

## Discussion

This study used the MSM to simultaneously estimate the short-term and long-term effects for patients with KF and patients with a kidney transplant. For comparison reasons, we also conducted the CPH model, which included time-varying components for the modality variable. The survival probabilities for patients with KF varied across different demographics and SDOH.

Intuitively, the ORs within the MSM indicated a decrease in long-term survivability when age increased. On the other hand, the HRs within the MSM indicated a decrease in short-term survivability for those 18 years or older compared with those younger than 18 years. The MSM results indicated that the HR did not become higher than those younger than 18 years until they were 50 or 60 years old or above.

Based on the CPH model results, White patients had the highest HR (the lowest survivability) compared with other races. The short-term effects of the MSM affirmed this result. In other words, the minority groups tended to have better survivability even after accounting for factors such as socioeconomic status, comorbid condition, etc. This appeared counterintuitive. A previous study described this phenomenon as a survival paradox.^19^ Many suggestions, eg, cultural differences and survival bias, were proposed to support this paradox. However, no exact reason for this phenomenon was found,^33^ and none of the suggestions were quantified. Conversely, the long-term effects of the MSM challenged this inconsistency. We found that American Indians had the lowest long-term survivability, followed by Black patients and then White patients. In other words, American Indian and Black patients had a higher probability of being within the group characterized as having shorter survivability. Multiple studies report that minorities with KF have a drawback when it comes to kidney transplantation.^34^, ^35^, ^36^ They proposed many reasons, eg, the lack of suitable transplants for minority groups due to medical factors. In addition, when we examined the average age at the time of first enrollment in an KRT, the average age of American Indians (57.6 years) was the lowest among all other races, followed by the groups of other races (58.5 years), Black (58.9 years), NH/PI (59.1 years), Asian (63.5 years), and finally White (64.6 years). This implied that White patients started dialysis 7 years later than American Indian patients, on average. The same reasoning might be applied when considering the average age at first enrollment in an KRT across Hispanic groups or patients with different insurance statuses. Non-Hispanic patients had an average dialysis start age (63.5 years) about 5 years older than Hispanic patients (58.4 years). Based on both the CPH model and MSM, Hispanic patients, therefore, had higher long-term survivability. Similarly, the average age for patients without insurance (49.3 years) was the lowest compared with patients with other types of insurance. In contrast, those within the groups Medicare and employer (69.3 years), Medicare only (69.9 years), and multiple otherwise (72.6 years) had the top 3 highest average ages. This likely explains the higher survivability for patients without insurance. Further discussion on the distribution of age across insurance categories may be found in the supplementary material.

Comorbidity played an important role in the survival probability of patients on KRT. Many results related to comorbidity were intuitive. For instance, the patients with diabetes and the patients with higher Liu comorbidity index tended to have higher HRs and ORs, according to the CPH model and MSM. Similarly, the patients with the inability to ambulate, inability to transfer, needed assistance with their daily activities, had amputations, or were institutionalized, each had higher HRs. Patients with toxic nephropathy had lower HRs according to both the CPH model and MSM. The MSM showed that patients with an inability to transfer, needed assistance with their daily activities, or who were institutionalized each had higher long-term survivability. Although they might be a few years older than their counterparts, it was possible that their disability benefits might allow them to access kidney transplants and thus explain the higher survivability.

According to the MSM, patients with peritoneal dialysis had a lower HR compared with individuals with hemodialysis and yet had higher OR in the MSM. These results suggested that those patients receiving peritoneal dialysis had a higher short-term survivability; on the contrary, patients who underwent hemodialysis had a higher long-term survivability. This is further supported by results related to the time-dependent modality variable within the CPH model (shown in Fig 4). A study found that those on peritoneal dialysis had higher survivability for the first 2 years. Conversely, hemodialysis resulted in similar or higher survivability once beyond these 2 years.^37^ These conclusions supported the results of the MSM in terms of short-term and long-term survivability and were similar to the CPH model results. Additionally, the MSM results reveal that patients with at least one transplant had a higher long-term survivability.

As expected, patients with an illicit drug dependence had a higher HR than those without. However, the ORs within the MSM then counterintuitively displayed that those with an illicit drug dependence had better long-term survivability. This oddity might exist because those with illicit drug dependence had an average age of just 48.9 years, versus those without the dependence had an average age of 63.0 years. Further, the results reveal that the employed patients had both lower HR and OR were intuitive. Also, while the HR results of patients living in rural areas were mixed compared with those patients from urban areas, the OR results of the MSM intuitively showed that patients from rural areas had lower long-term survivability. Interestingly, patients living in any region had both higher HRs and ORs compared with individuals from the Western region. These results were consistent with the 2020 Annual Report of USRDS.^38^

This study included various limitations. The assumption of proportional hazards within the CPH model might be alleviated by using an MSM.^39^ Furthermore, adjusting for more SDOH, social constructs, and medical factors would further improve results because the current model developed was most likely an underspecified model. In conclusion, various factors, including demographics and SDOH, affected the HRs and ORs of all-cause mortality for patients with KF. The MSM allowed inferences on both short-term and long-term effects of all covariates without directly incorporating time-varying components within the survival analysis, as was done in the CPH model. Therefore, the MSM would enable health care providers to better address the short-term and long-term effects of each factor on patients with KF and eventually make more data-driven KF treatment decisions.
