# Supplementary material for: Estimating Short-Term and Long-Term Survival for Patients With Kidney Failure Using a Mixture Survival Model
Source: Kidney Med. 2025 Dec 26;8(3):101232. doi: 10.1016/j.xkme.2025.101232 (PMC12874817; doi:10.1016/j.xkme.2025.101232)
Supplement: Supplementary File (PDF) — Figure S1; Table S1. [file mmc1.pdf]

Estimating Short-Term and Long-Term Survival for Patients with Kidney Failure Using a Mixture Survival Model

Supplementary Material

Nathan Meyer, Maxwell Donelan, Hossein Moradi Rekabdarkolaee, Brandon M. Varilek, Surachat Ngorsuraches, Patti Brooks, Jerry Schrier, and Semhar Michael*

Table of Contents

- Supplemental Section 1. Insurance categories in the USRDS data for patients with kidney failure
  - Figure S1. Cumulative barplot across age groups for each level of the insurance variable.
  - Table S1. AIC and BIC values of a Cox regression model with varying options for the insurance covariate.
- Supplemental Section 2. Model diagnostics and computational strategies
- Supplementary References

Supplemental Section 1. Insurance categories in the USRDS data

We performed variable selection on the Cox regression model using two separate criteria: Akaike information criterion (AIC) and Schwarz's Bayesian criterion (BIC). These criteria were used along with one of the three methods: forward, backward, or both step-wise variable selection. When considering AIC, the step function suggested using a model that includes all variables, regardless of which of the three methods was used. When using BIC with any of the three methods, it is recommended to remove the Rurality variable. Note that these statements are both true when taking into account either the entire dataset or only those on dialysis. Interestingly, age was chosen first when performing forward selection in any setting. When considering both the dialysis and transplant population, age is then followed by transplant status, Liu comorbidity index, insurance, and then race. When taking into account only those on dialysis, age is preceded by Liu's comorbidity index, race, Hispanic status, inability to ambulate, and then insurance. Note that, as expected, the last covariate chosen during forward selection using AIC was rurality.

The many insurance options discussed in the main text allowed for many different ways to create the final insurance covariate. Furthermore, insurance was among the first few variables selected, indicating its importance. Thus, we explored other options for making the insurance covariate using the 34 unique combinations of insurance status. Table 1 displays several new options for creating the insurance variable along with the corresponding AIC and BIC values after fitting a model using Cox regression on either the dialysis and transplant population or the only dialysis population. All options for how the insurance covariate was constructed are abbreviated within the table such that each, in order, is expanded as: (1) the primary one used throughout the main text; (2) same as option one with the exception that the groups of Medicare and none are combined; (3) private represents any person with employer insurance, public represents any person with Medicare, Medicaid, or DVA but not employer insurance, other and none is as defined in the dataset; (4) same as option three but with public and none combined; (5) all 34 possible categories found within the dataset as discussed in the main text; (6) using only categories from option five that have a sample size greater than 0.1% of the dataset prior to cleaning; (7) same as option six but sample size must be larger than 0.7%; (8) same as option six but sample size must be larger than 1%; (9) same as option seven but Employer-only, Medicaid-only, and DVA-only were combined.

The first option shown in the table was ultimately chosen for the inferences given in the main text. The paper by Nee et al.[1] used a similar method for creating an insurance covariate using the USRDS data. On the other hand, research about the survival of patients with kidney failure by Brown-Tortorici et al.[2] mentioned categorizing an insurance variable by combining Medicare and Medicaid into one category. Furthermore, a study by Baptiste et al.[3] on KF patients in relation to COVID-19 considered three insurance categories: public, private, and none. Lastly, research about survival analysis of patients with KF using the USRDS by Jurkovitz et al.[4] similarly used public, private, and none as categories. These academic works display why options three and four may be considered. Furthermore, since U.S. law allows for easy access to Medicare immediately after kidney failure,,[5] combining the insurance category none with either Medicare or public insurance within options two and four, respectively, is justifiable. Note that the top few best options in terms of AIC or BIC are relatively close in value within each of the four columns. Also, we consider only the dialysis group of individuals when fitting the final Cox regression model since it would be inappropriate otherwise, as stated in the main text. Lastly, option one is tied for lowest (with rounding) when considering BIC and the dialysis-only population. Overall, option one appears to be an adequate representation of the insurance information.

*Correspondence: semhar.michael@sdstate.edu

The barplots given in Figure S1 represent the insurance categories used in our analysis and their corresponding age distribution. We can observe some similar insurance categories based on their age group distributions. For example, the age distribution of people who have employer only, Medicaid only, or no insurance are very similar. There appear to be younger people with these insurance types than the other categories (heavy left tails).

Table S 1. AIC and BIC values of a Cox regression model with varying options for the insurance covari-ate. “Both” indicates the model was computed using both the transplant and dialysis population whereas “Dialysis” indicates the model was computed using only the dialysis population. Note that the best (lowest) three values for each column are emphasized.

Setting	AIC Both	BIC Both	AIC Dialysis	BIC Dialysis
(1) Used	42,502,339	*42,502,940*	40,552,045	*40,552,632*
(2) Used - MDCR and none	42,506,741	42,507,330	40,556,087	40,556,661
(3) Private, public, other, none	42,507,232	42,507,772	40,556,531	40,557,057
(4) Private, public, other	42,511,439	42,511,966	40,560,370	40,560,884
(5) All 34 categories	*42,502,225*	42,503,132	*40,551,941*	40,552,833
(6) > 0.1%	*42,502,231*	*42,502,930*	*40,551,947*	*40,552,632*
(7) > 0.7%	42,502,322	42,502,947	40,552,037	40,552,648
(8) > 1%	42,502,475	42,503,076	40,552,172	40,552,759
(9) > 0.7% - Emp, MDCD, DVA	*42,502,320*	*42,502,933*	*40,552,035*	*40,552,634*

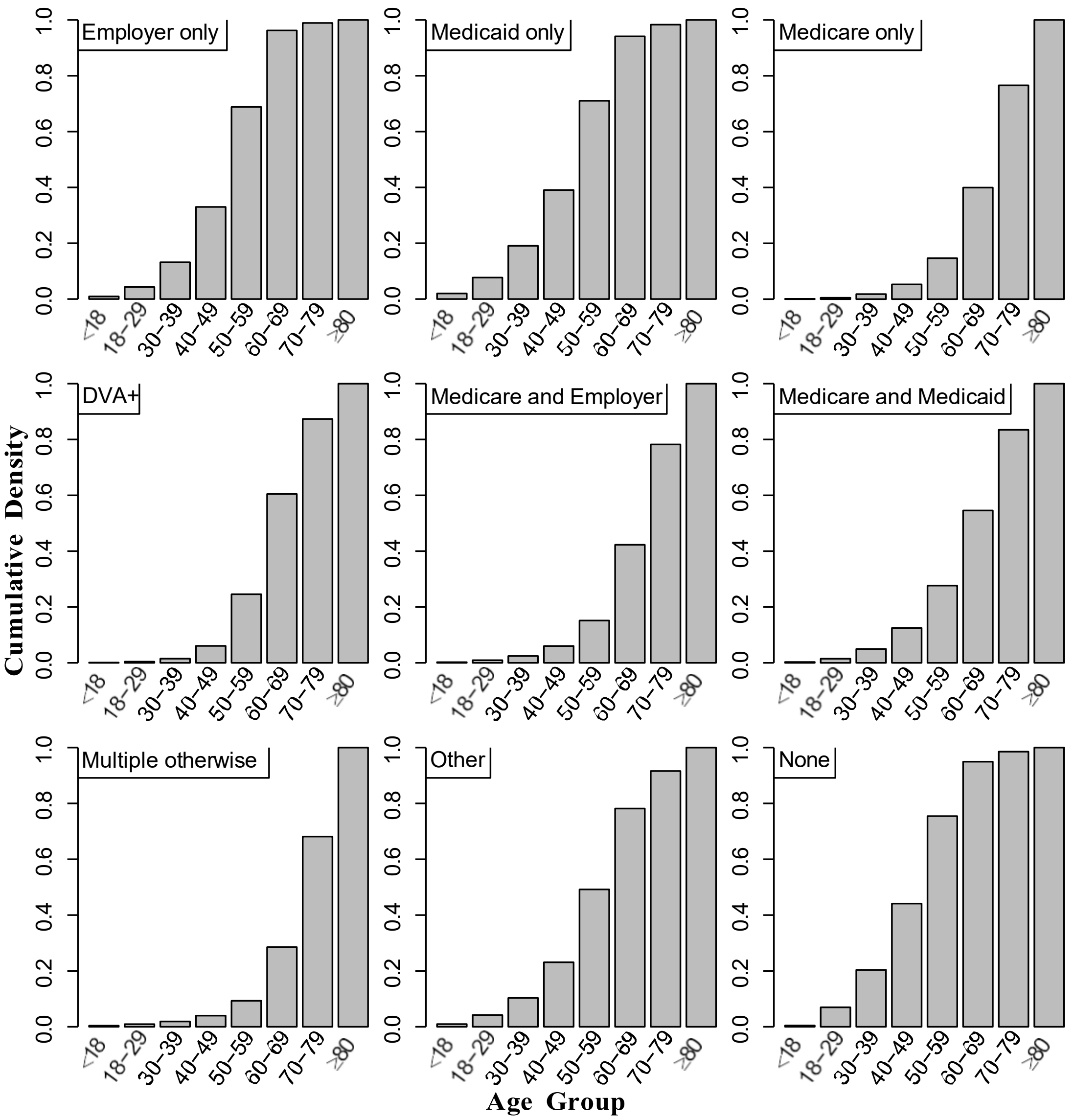

Figure S 1 . Cumulative barplot across age group for each level of the insurance variable.

Supplemental Section 2. Model diagnostics and computational strategies

This section discusses the appropriateness of the mixture survival model using biological and empirical evidence and the computational strategies employed to fit the mixture model via the expectation-maximization (EM) algorithm to the data of such size.

Farewell[6] discusses the importance of only using mixture survival models when strong evidence to do so arises. Sy and Taylor[7] gives several indicators for when a dataset could benefit from applying a mixture survival model. These indicators may be summarized into five concepts to be then applied to a dataset: biological evidence, empirical evidence, large sample, long-term follow-up, and non-excessive censoring. As said within the main text, individuals who have received a successful transplant would no longer be on dialysis and have a greater survival. This would indicate biological evidence for using a mixture survival model. Empirical evidence also supports the use of such a model, as about 11.0% of the approximately 2.2 million people within the dataset have had at least one transplant, and this would contain successful transplant recipients. It is also suggested that we should have a large sample to continue with fitting a mixture survival model; this is satisfied as most would consider about 2.2 million observations a large amount in the context of survival analysis. The USRDS dataset also has long-term follow-up time as the data presented was collected over the span of about 20 years. Lastly, a moderate amount of almost 30.0% of the close to 2.2 million people were censored (individuals who were not recorded as experiencing the event of interest). Each of the previous points motivates the need for a mixture survival model to be considered.

To fit the mixture model to the given data, all code was written in the R Statistical Software version 4.3.2.[8] and will be available on the project's GitHub page. Preparation of the data, including cleaning and filtering as described in the main text, is done by making use of the *R* package *dplyr* from Wickham et al.[9] The packages *Hmisc* from Harrell Jr[10] and *gtsummary* from Sjoberg et al.[11] were then used to create summary tables. The well-known package *survival* from Therneau[12] was used to fit a Cox PH regression model for comparison with the mixture survival model.

For the mixture survival model, due to the size of the USRDS dataset, the already developed package *smcure* from Cai et al.[13] could not be used as written. Therefore, the code was rewritten to both fit the model to the data and provide confidence intervals for parameter estimates through the bootstrap approach within a reasonable amount of time. The rewritten code had abilities such as allowing initial values as input and performing parallel computing to allow efficient computation and timely convergence. Since the EM algorithm is computationally expensive, sequential computing during the bootstrap sampling is infeasible for large datasets. Thus, parallel computing algorithms given in the package *doParallel* from Corporation and Weston[14] were used to quicken the bootstrap sampling when fitting the model. A function was written to allow multiple cores to be used during the bootstrap procedure. With this, the high-performance computing (HPC) services at South Dakota State University were used to access many computer cores simultaneously, along with accessing high amounts of random access memory. Using parallel computing with these HPC services, the median time across each computer node accessing multiple cores for 100 bootstrap samples on the mixture model was about 42.38 hours, making sequential computing infeasible. Therefore, parallel computing was a necessary adaptation of the function when considering the USRDS dataset.

Furthermore, as discussed in the paper by Michael and Melnykov,[15] it is well known that initialization is a crucial step for parameter estimation procedures using the EM algorithm. The inclusion of allowing initial values often allowed us to start the EM algorithm at a further step in the process, compared to using the default initial values and finding a trustworthy parameter estimate. This sped up the process of finding coefficient estimates when making small changes to the model (*i.e.* adding or removing covariates). In our implementation, we used a likelihood based on Breslow's approach similar to the *smcure* package. Lastly, the stopping criterion for the EM algorithm was set to be the sum of the squared changes of the incidence, latency, and survival estimates with a margin of error below 1e-07. Finally, to represent the results of the model fit, a visualization tool (forest plot) was utilized to present the coefficient estimates and confidence intervals using the *metafor* package from Viechtbauer.[16]

Financial Disclosure

The authors declare that they have no relevant financial interests.

Support

The research reported in this paper was supported by South Dakota State University, AIM-AHEAD Coordinating Center, award number OTA-21-017, and was, in part, funded by the National Institutes of Health Agreement No. 1OT2OD032581.

Disclaimer

The work is solely the responsibility of the authors and does not necessarily represent the official view of AIM -AHEAD or the National Institutes of Health. The data reported here have been supplied by the United States Renal Data System (USRDS). The interpretation and reporting of these data are the responsibility of the author(s) and in no way should be seen as an official policy or interpretation of the U.S. government.

Data Availability

Access to USRDS data is limited to researchers and institutions with approved Data Use Agreements and will not be released. The relevant code used to generate the results presented within this paper will be posted to GitHub.

Supplementary References

[1] Robert Nee, John S Thurlow, Keith C Norris, Christina Yuan, Maura A Watson, Lawrence Y Agodoa, and Kevin C Abbott. Association of race and poverty with mortality among nursing home residents on maintenance dialysis. *Journal of the American Medical Directors Association*, 20(7):904–910, 2019. https://doi.org/10.1016/j.jamda.2019.02.013.

[2] Amanda R Brown-Tortorici, Yoko Narasaki, Amy S You, Keith C Norris, Elani Streja, Rene Amel Peralta, Yalitzi Guerrero, Andrea Daza, Ria Arora, Robin Lo, Tracy Nakata, Danh V Nguyen, Kamyar Kalantar-Zadeh, and Connie M Rhee. The interplay between dietary phosphorous, protein intake, and mortality in a prospective hemodialysis cohort. *Nutrients*, 14(15):3070, 2022. https://doi.org/10.3390/nu14153070.

[3] Claire S Baptiste, Esther Adegbulugbe, Divya Shankaranarayanan, Zahra Izzi, Samir Patel, Rasha Nakity, Richard L Amdur, and Dominic Raj. Prevalence and predictors of outcomes among esrd patients with covid-19. *BMC nephrology*, 24(1):67, 2023. https://doi.org/10.1186/s12882-023-03121-5.

[4] Claudine T Jurkovitz, Suying Li, Keith C Norris, Georges Saab, Andrew S Bomback, Adam T Whaley -Connell, Peter A McCullough, and Keep Investigators. Association between lack of health insurance and risk of death and esrd: results from the kidney early evaluation program (keep). *American journal of kidney diseases*, 61(4):S24–S32, 2013. https://doi.org/10.1053/j.ajkd.2012.12.015.

[5] U.S. Centers for Medicare and Medicaid Services, 2024. End-Stage Renal Disease (ESRD). Retrieved 2024-06-17, from https://www.medicare.gov/basics/end-stage-renal-disease.

[6] Vernon T Farewell. Mixture models in survival analysis: Are they worth the risk? *Canadian Journal of Statistics*, 14(3):257–262, 1986. https://doi.org/10.2307/3314804.

[7] Judy P Sy and Jeremy MG Taylor. Estimation in a cox proportional hazards cure model. *Biometrics*, 56(1):227–236, 2000. https://www.jstor.org/stable/2677126.

[8] R Core Team. *R: A Language and Environment for Statistical Computing*. R Foundation for Statistical Computing, Vienna, Austria, 2023. https://www.R-project.org/.

[9] Hadley Wickham, Romain Fran¸cois, Lionel Henry, Kirill M u¨ ller, and Davis Vaughan. *dplyr: A Grammar of Data Manipulation*, 2023. R package version 1.1.4. https://CRAN.R-project.org/package=dplyr.

[10] Frank E Harrell Jr. *Hmisc: Harrell Miscellaneous*, 2024. R package version 5.1-2. https://CRAN.R-project.org/package=Hmisc.

[11] Daniel D. Sjoberg, Karissa Whiting, Michael Curry, Jessica A. Lavery, and Joseph Larmarange. Reproducible summary tables with the gtsummary package. *The R Journal*, 13:570–580, 2021. https://doi.org/10.32614/RJ-2021-053.

[12] Terry M Therneau. *A Package for Survival Analysis in R*, 2023. R package version 3.5-5. https://CRAN.R-project.org/package=survival.

[13] Chao Cai, Yubo Zou, Yingwei Peng, and Jiajia Zhang. *smcure: Fit Semiparametric Mixture Cure Models*, 2022. R package version 2.1. https://CRAN.R-project.org/package=smcure.

[14] Microsoft Corporation and Steve Weston. *doParallel: Foreach Parallel Adaptor for the 'parallel' Package*, 2022. R package version 1.0.17. https://CRAN.R-project.org/package=doParallel.

[15] Semhar Michael and Volodymyr Melnykov. An effective strategy for initializing the em algorithm in finite mixture models. *Advances in Data Analysis and Classification*, 10:563–583, 2016. https://doi.org/10.1007/s11634-016-0264-8.

[16] Wolfgang Viechtbauer. Conducting meta-analyses in R with the metafor package. *Journal of Statistical Software*, 36(3):1–48, 2010. https://doi.org/10.18637/jss.v036.i03.
